# Pyrrolizidine Alkaloid-Induced Hepatotoxicity Associated with the Formation of Reactive Metabolite-Derived Pyrrole–Protein Adducts

**DOI:** 10.3390/toxins13100723

**Published:** 2021-10-13

**Authors:** Jiang Ma, Mi Li, Na Li, Wood Yee Chan, Ge Lin

**Affiliations:** 1 School of Biomedical Sciences, Faculty of Medicine, The Chinese University of Hong Kong, Hong Kong 4054577, China; majoriea@163.com (J.M.); limi@tsinghua.edu.cn (M.L.); nali9898@hotmail.com (N.L.); wy-chan@cuhk.edu.hk (W.Y.C.); 2 School of Traditional Chinese Medicine, Guangdong Pharmaceutical University, Guangzhou 510000, China

**Keywords:** pyrrolizidine alkaloids, pyrrole–protein adducts, pyrrolizidine alkaloid-containing herbs, toxicity screening and ranking, *Crotalaria sessiliflora*, *Gynura japonica*

## Abstract

Pyrrolizidine alkaloids (PAs) with 1,2-unsaturated necine base are hepatotoxic phytotoxins. Acute PA intoxication is initiated by the formation of adducts between PA-derived reactive pyrrolic metabolites with cellular proteins. The present study aimed to investigate the correlation between the formation of hepatic pyrrole–protein adducts and occurrence of PA-induced liver injury (PA-ILI), and to further explore the use of such adducts for rapidly screening the hepatotoxic potency of natural products which contain PAs. Aqueous extracts of *Crotalaria sessiliflora* (containing one PA: monocrotaline) and *Gynura japonica* (containing two PAs: senecionine and seneciphylline) were orally administered to rats at different doses for 24 h to investigate PA-ILI. Serum alanine aminotransferase (ALT) activity, hepatic glutathione (GSH) level, and liver histological changes of the treated rats were evaluated to assess the severity of PA-ILI. The levels of pyrrole–protein adducts formed in the rats’ livers were determined by a well-established spectrophotometric method. The biological and histological results showed a dose-dependent hepatotoxicity with significantly different toxic severity among groups of rats treated with herbal extracts containing different PAs. Both serum ALT activity and the amount of hepatic pyrrole–protein adducts increased in a dose-dependent manner. Moreover, the elevation of ALT activity correlated well with the formation of hepatic pyrrole–protein adducts, regardless of the structures of different PAs. The findings revealed that the formation of hepatic pyrrole–protein adducts—which directly correlated with the elevation of serum ALT activity—was a common insult leading to PA-ILI, suggesting a potential for using pyrrole–protein adducts to screen hepatotoxicity and rank PA-containing natural products, which generally contain multiple PAs with different structures.

## 1. Introduction

**Figure 1:**
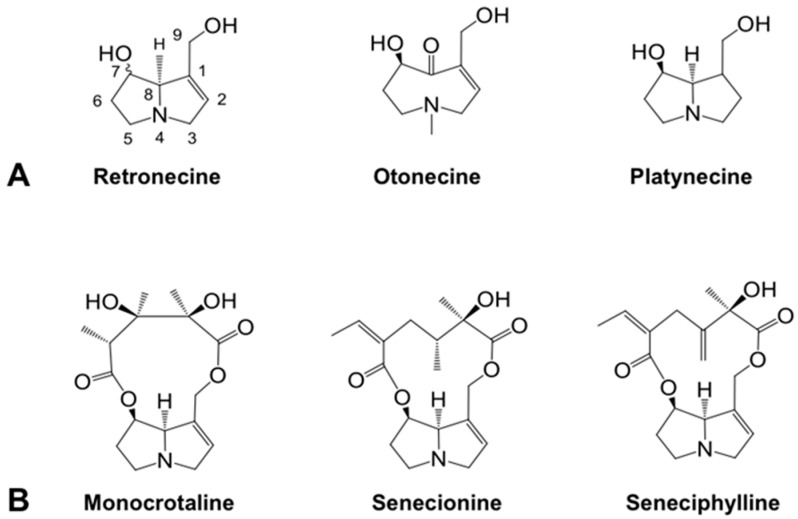
Chemical structures examined in the present study: (**A**) three PA necine bases; (**B**) three PAs (monocrotaline, senecionine and seneciphylline).

**Figure 2:**
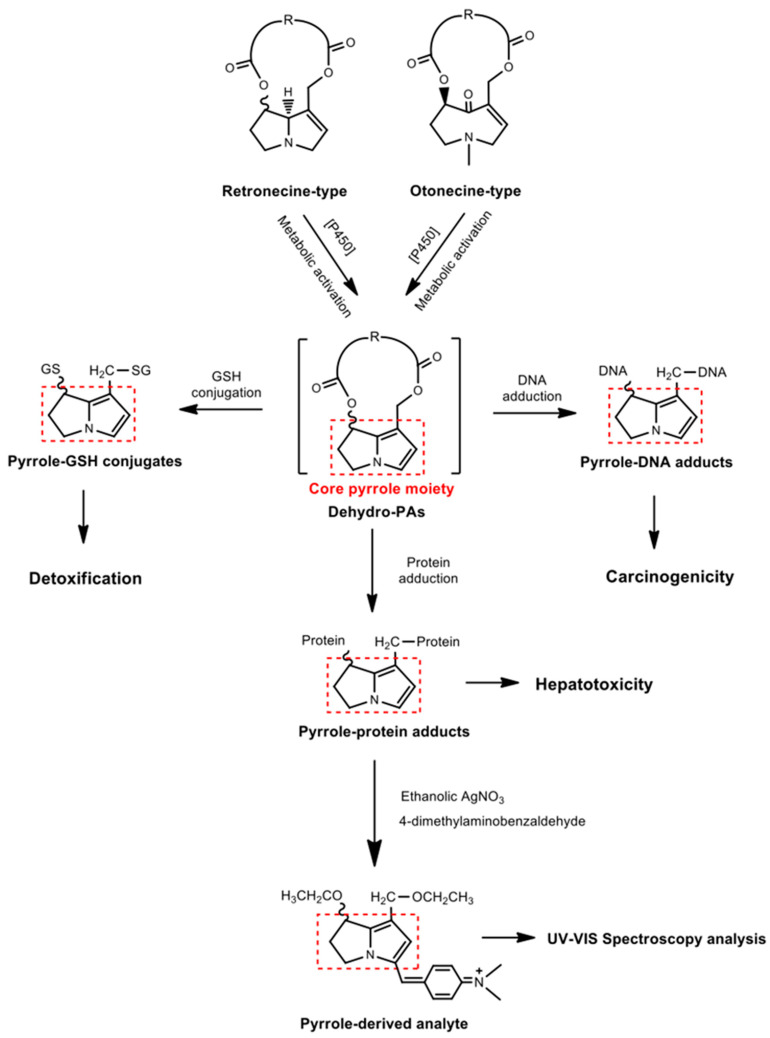
Metabolic activation of retronecine-type and otonecine-type PAs to form reactive dehydro-PAs which react with GSH, DNA, and proteins to generate PA-derived adducts. The subsequent Ehrlich reaction of pyrrole–protein adducts produces a detectable pyrrole–derived analyte.

Pyrrolizidine alkaloids (PAs) are widely present in about three percent of flowering plants, these belong to twelve different families. Boraginaceae, Asteraceae and Fabaceae contain the most toxic PAs [1,2,3]. To date, over 660 PAs which are present as free-base PAs and PA *N*-oxides have been identified in over 6000 plant species, and about half of them are hepatotoxic [1,2,3]. PAs are probably the most common phytotoxins presently affecting livestock, wildlife, and humans [1,2,3,4,5]. PAs are esters containing a necine base and an esterifying acid (Figure 1). Based on the structure of the necine base (Figure 1A), PAs are classified into retronecine-type (including its C7-stereoisomer), otonecine-type, and platynecine-type PAs. Retronecine-type and otonecine-type PAs with a double bond in unsaturated necine base are hepatotoxic and carcinogenic [1,2,3,6]. Platynecine-type PAs, having a saturated necine base, are generally considered as non-toxic [7]. Toxic PAs require metabolic activation, catalyzed by cytochrome P450 isozymes in the liver, to form dehydropyrrolizidine alkaloids (dehydro-PAs) [1,2,3,6]. Dehydro-PAs are biologically reactive and rapidly bind to cellular proteins and DNA to form pyrrole–protein adducts and pyrrole–DNA adducts, leading to hepatotoxicity and carcinogenicity (Figure 2) [1,2,3,4,5,6,8,9,10,11,12]. Besides, dehydro-PAs can also react with the reduced form of glutathione (GSH) to generate pyrrole–GSH conjugates, which are rapidly excreted out of the body [13,14]. Consequently, the competitive interaction of dehydro-PAs with hepatic macromolecules (intoxication pathway) or GSH (detoxification pathway) determines the severity of PA-ILI [14,15,16,17].

Depending on the dose and duration of exposure, all unsaturated PAs have the potential to cause PA-ILI to different severities. These include an instance of severe liver damage named hepatic sinusoidal obstruction syndrome (HSOS)—previously called hepatic veno-occlusive disease (HVOD)—which is associated with hepatomegaly, ascites, hyperbilirubinaemia, and hemorrhagic necrosis [18,19,20]. The earliest human case of PA-induced HSOS was reported in 1920 and was associated with the ingestion of PA-contaminated wheat [21]. Since then, several outbreaks of PA intoxication have been reported in various countries, including China, where ingestion of PA-producing herbs, particularly *Gynura japonica*, has been reported as a main cause of PA-induced HSOS [19,21,22,23,24,25,26]. Moreover, due to the lack of specific and confirmative methods of diagnosing PA intoxication, the true incidence of human PA-ILI may be much higher. 

In recent years, based on the biochemical mechanisms underlying PA-ILI, our group has detected pyrrole–protein adducts in the livers and blood of PA-treated rodents, and in the blood of PA-ILI patients, caused by ingestion of PA-producing herbs [2,6,9,24,27]. The determination of blood pyrrole–protein adducts has been successfully applied for confirmative clinical diagnosis of PA-ILI patients [19,24,27,28]; nevertheless, the correlation between pyrrole–protein adducts formed by different PAs and the severity of PA-ILI has not been well characterized. In the present study, using rats orally treated with water extracts of two PA-producing herbs, the correlation between the formation of pyrrole–protein adducts and the severity of PA-ILI was evaluated. The two PA-producing herbs used in the study were *Crotalaria sessiliflora* L., containing only one PA (monocrotaline), and *Gynura japonica* (Thunb.) Juel, containing predominately two PAs (senecionine and seneciphylline) (Figure 1B). The results demonstrated that both PA-producing herbal extracts developed liver damage in a dose-dependent manner; additionally, and more interestingly, the elevation of ALT activity correlated well with the formation of hepatic pyrrole–protein adducts, regardless of the structures of different PAs present in the herbal extracts. Our findings suggested a good correlation between the formation of hepatic pyrrole–protein adducts and the severity of PA-ILI; thus, hepatic pyrrole–protein adducts have the potential to be developed as a biomarker for rapid evaluation of the potential toxicity and severity of PA-ILI caused by PA-containing herbs and PA-contaminated food stuffs.

## 2. Results 

### 2.1. Effects of C. sessiliflora Extract on Rats

#### 2.1.1. Effect on Serum ALT Activity

Serum ALT activity reflects damage to hepatocytes and is commonly used as a highly sensitive biomarker for clinical and research laboratory tests of liver injury [29]. Therefore, in the present study, serum ALT activity was employed as a primary biomarker to assess the severity of hepatotoxicity induced by PAs. As the results summarize in Table 1, *C. sessiliflora* extract treatment significantly increased serum ALT activity in rats at 24 h post-dosing, when the dose was increased to 1.09 g extract/kg (equivalent to 0.70 mmol/kg of monocrotaline, calculated by the content of monocrotaline in *C. sessiliflora* extract, as indicated in Materials and Methods section) (*p* < 0.01) or above, compared with to the corresponding vehicle control group. Furthermore, pure monocrotaline (0.80 mmol/kg) treatment also caused a significant increase in serum ALT activity, within the range induced by monocrotaline-containing *C. sessiliflora* extract, with doses equivalent to monocrotaline doses of 0.77–0.96 mmol/kg (Table 1). These results demonstrated that the *C. sessiliflora* extract-induced liver damage was primarily caused by the monocrotaline which was present in the extract. The results clearly showed that, as indicated by the elevated serum ALT activity measured at 24 h after oral administration, monocrotaline in *C. sessiliflora* extract induced liver damage in a dose-dependent manner. 

#### 2.1.2. Effect on Hepatic GSH Level 

It has been established that hepatic GSH reacted with dehydro-PA to form pyrrole–GSH conjugates, which were readily excreted from the body (Figure 2). Exposure to PAs stimulated GSH synthesis and thereby increased hepatic GSH levels; therefore, such increase is considered as a self-defense mechanism in preventing or reducing hepatotoxicity. However, GSH depletion would occur if a significant amount of dehydro-PA formed very rapidly, exhausting the available GSH, without allowing time for new GSH synthesis in the liver, and thus this self-defense system would be overwhelmed [30]. In the present study, when compared with the corresponding control groups, significant increases in hepatic GSH level were observed in most of the *C. sessiliflora* extract treated groups (Table 1), suggesting that the treatment doses of monocrotaline in the *C. sessiliflora* extract were not high enough to cause GSH depletion. This result is in a good agreement with previous studies, which also show an increase in hepatic GSH level 24 h after oral administration of monocrotaline [13,31].

#### 2.1.3. Effect on Liver Histological Changes

**Figure 3:**
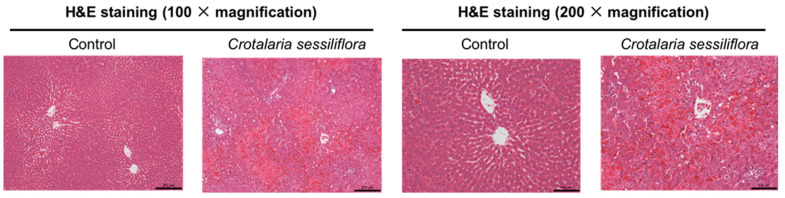
Representative H&E-stained liver sections obtained from rats in the control and the *Crotalaria sessiliflora* water extract-treated (1.2 g/kg) groups.

Liver sections obtained from rats treated with *C. sessiliflora* extract, or monocrotaline, were examined using hematoxylin–eosin (H&E) staining (Figure 3). It is well established that PAs can induce characteristic symptoms such as hemorrhage, coagulative necrosis of hepatocytes, and fibrosis [18,30,32]. Thus, in the present study, hemorrhage and coagulative necrosis were selected as parameters to evaluate PA-induced hepatotoxicity, while fibrosis was not examined because it mainly occurs after chronic exposure to PAs [32]. No lesions were observed in the livers obtained from the control rats, while the rats receiving *C. sessiliflora* extract, starting from monocrotaline equivalent dose of 0.50 mmol/kg and pure monocrotaline (0.80 mmol/kg), developed hemorrhages and coagulative necrosis in a dose-dependent manner (Table 2). The results also suggested that coagulative necrosis of hepatocytes was typical in the liver and started to occur at the dose of 0.50 g/kg *C. sessiliflora* extract (monocrotaline 0.32 mmol/kg), well before hemorrhage was observed. Hemorrhage occurred firstly in areas around central veins, at doses higher than 0.78 g/kg *C. sessiliflora* extract (equivalent to monocrotaline dose of 0.50 mmol/kg). The histological results clearly demonstrated a dose-dependent liver injury caused by monocrotaline and monocrotaline-containing *C. sessiliflora* extract (Table 2).

#### 2.1.4. Effect on Formation of Hepatic Pyrrole–Protein Adducts 

**Figure 4:**
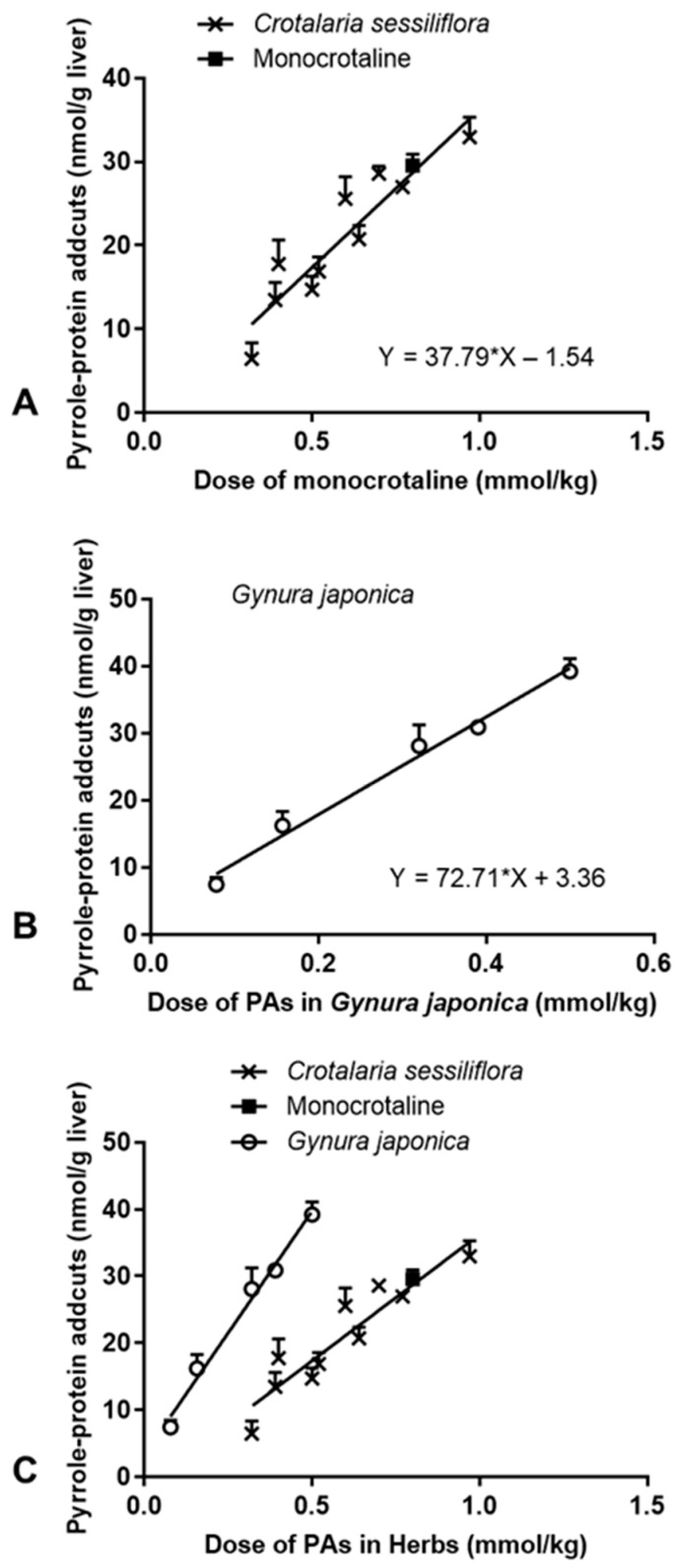
Correlation between the amount of pyrrole–protein adducts formed in the livers of PA-treated rats and the oral dosage of PA (monocrotaline) in: *Crotalaria sessiliflora* (**A**); *Gynura japonica* (**B**); in both herbal plants (**C**). Data are expressed as mean ± SEM (*n* = 5).

The PA-induced action mechanism of hepatotoxicity has been extensively investigated, and previous studies have already shown that the formation of hepatic pyrrole–protein adducts is directly related to PA-ILI [6,9,24]. In the present study, hepatic pyrrole–protein adducts were detected in all liver specimens obtained from rats treated with *C. sessiliflora* extract or pure monocrotaline, but not in the livers of control rats (Table 1). The formation of hepatic pyrrole–protein adducts increased in a dose-dependent manner. A correlation with a good linearity (*r*^2^ = 0.86) was observed between the dose of monocrotaline given in a pure form, or present in *C. sessiliflora* extracts, and the amount of hepatic pyrrole–protein adducts (Figure 4A). The results indicated that the formation of hepatic pyrrole–protein adducts was directly correlated with the dose of monocrotaline, either in a pure form or present in the *C. sessiliflora* extracts containing monocrotaline. 

### 2.2. Effects of G. japonica Extract on Rats

To further explore the possibility of establishing hepatic pyrrole–protein adducts as a biomarker, directly relating to the dose and the induced hepatotoxicity of different PAs, various doses of *G.* *japonica* extract containing two PAs were orally given to rats and biological samples were collected at 24 h after treatment. These samples were analyzed for serum ALT activity, hepatic GSH level, and hepatic pyrrole–protein adducts. Because *G.* *japonica* contains two toxic PAs, namely senecionine and seneciphylline, the hepatotoxicity observed would be the overall effect of both PAs; thus, the PA dose was calculated based on the total amount of the two PAs in the extract. The results showed that, similarly to the *C.* *sessiliflora* extract treatment, *G.* *japonica* extract also induced hepatotoxicity in the treated rats in a dose-dependent manner (Table 1). Moreover, hepatic pyrrole–protein adducts were also produced in a dose-dependent manner, with a good linear correlation (*r*^2^ = 0.99) between the total PA content in *G.* *japonica* extract and the amount of hepatic pyrrole–protein adducts (Figure 4B). Interestingly, when comparing the treatment of both herbal extracts, it was found that the linearity slope of the formation of hepatic pyrrole–protein adducts of *G.* *japonica* extract (72.71 ± 4.70) was significantly higher than that of *C.* *sessiliflora* extract (37.79 ± 5.48, *p* < 0.0001) (Figure 4C). This indicated a remarkably higher formation rate of hepatic pyrrole–protein adducts in the senecionine/seneciphylline-containing *G.* *japonica* extract treatment group than in the monocrotaline-containing *C.* *sessiliflora* extract treatment group.

### 2.3. Correlation between PA-ILI and Formation of Hepatic Pyrrole–Protein Adducts

**Figure 5:**
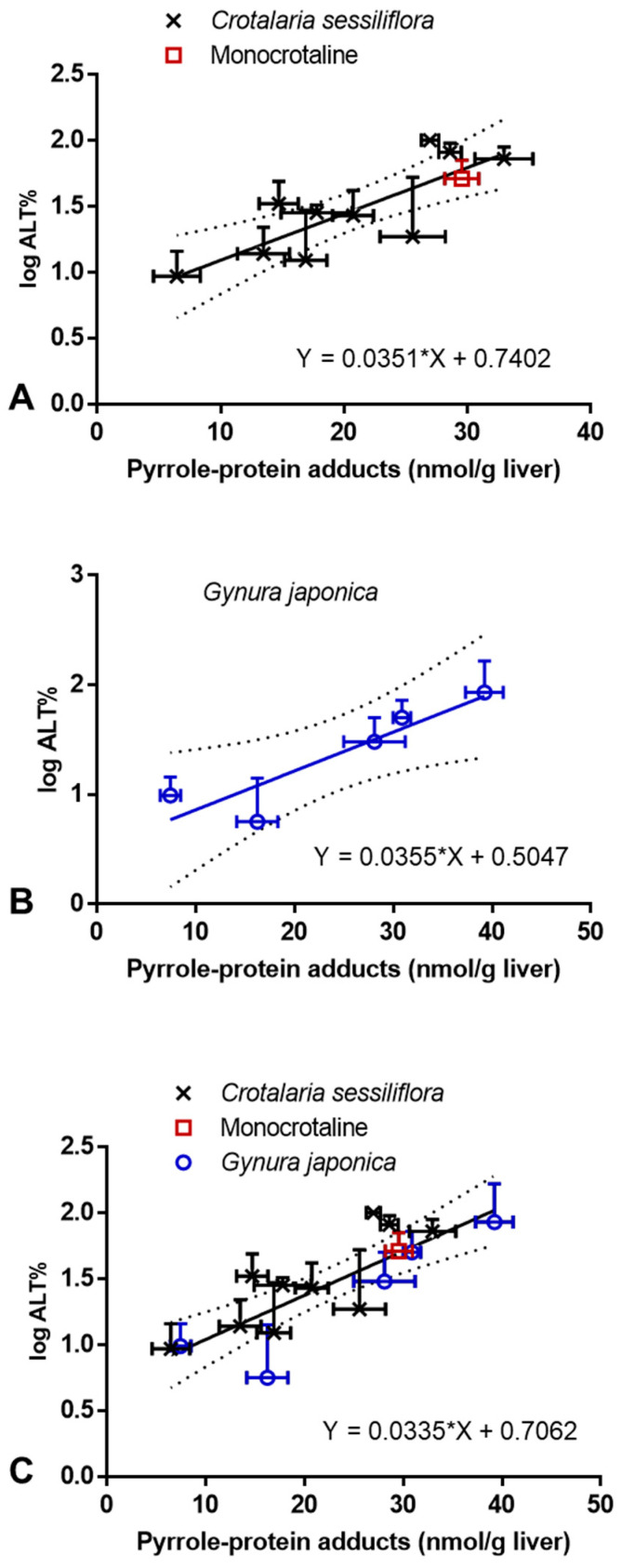
Correlations between the level of hepatic pyrrole–protein adducts and the log values of the percentage of elevated serum ALT activity in rats orally dosed with: *Crotalaria* *sessiliflora* extract and pure monocrotaline (**A**); *Gynura japonica* extract (**B**); both herbal extracts and pure monocrotaline (**C**). Log ALT% = log (ALT activity of PA-treated rat − ALT activity of control rat)/ALT activity of control rat × 100%. Data are expressed as mean ± SEM (*n* = 5).

The correlation between the severity of PA-ILI, as indicated by the elevation of serum ALT activity, and the formation of hepatic pyrrole–protein adducts was further examined. A good correlation was found from the plots, which showed a linear correlation between the formation of hepatic pyrrole–protein adducts and the log value of the percentage of elevated ALT activity in rats orally administered with *C. sessiliflora* extract or pure monocrotaline (Figure 5A) and *G. japonica* extract (Figure 5B). Furthermore, to compare the protein adducts formation in the three treatment groups, another plot (Figure 5C)—which combined all data obtained from the rats treated with one of the herbal extracts or monocrotaline—was obtained, with a best fit linearity of Y = 0.0335X + 0.7062 (*r*^2^ = 0.66). The comparison showed no significant difference among these three treatment groups (*G. japonica*: Y = 0.0355X + 0.5047; *C. sessiliflora*: Y = 0.0351X + 0.7402). Therefore, our findings further revealed that, regardless of different PAs given (either in the pure form or in PA-containing herbal extracts), the formation of hepatic pyrrole–protein adducts was directly correlated with the log value of the percentage of the elevated ALT activity. 

## 3. Discussion

PA-containing plants are among the most common poisonous plants affecting livestock, wildlife, and humans [1,2,3,4,5]. PAs exert hepatotoxicity via cytochrome P450-mediated metabolic activation, forming pyrrole–protein adducts which impair the biological activities of some functional proteins, resulting in liver damage [2,8]. To date, no quantitative study has yet been conducted between the formation of pyrrole–protein adducts and the degree of hepatotoxicity induced by pure PAs or PA-containing medicinal herbs. In the present study, using two PA-containing herbs (*C.* *sessiliflora* and *G.* *japonica*), we first demonstrated that PAs in these herbal extracts indeed induced PA-ILI, with typical symptoms of both hemorrhage and coagulative necrosis, in a dose-dependent manner in rats (Table 1 and Table 2). We then observed that there was a direct correlation between the amount of monocrotaline-containing *C.* *sessiliflora* extract, or pure monocrotaline, and the level of hepatic pyrrole-–protein adducts formed (Figure 4). We also observed a very good correlation between the elevation of serum ALT activity, a well-established indicator of liver injury, and the formation of hepatic pyrrole–protein adducts (Table 1 and Figure 5). Thus, it is speculated that hepatic pyrrole–protein adducts could be used as a biomarker to indicate the degree of hepatotoxicity induced by PAs (e.g., monocrotaline) and PA-containing medicinal herbs. To further explore the possibility of using hepatic pyrrole–protein adducts as a biomarker, *G.* *japonica* extract containing two PAs (senecionine and seneciphylline) was also tested in the rat model. A similar correlation was found in the *G.* *japonica* extract treatment group, suggesting that—regardless of structures of PAs and whether they were given in a pure form or as components of herbal extracts—the formation of hepatic pyrrole–protein adducts was directly related to the severity of PA-ILI (Figure 5). Furthermore, the formation rate of hepatic pyrrole–protein adducts depended upon the structure and the dose of PAs, and of course, the contents of PAs in herbal extracts (Figure 4). Thus, our results clearly suggest that hepatic pyrrole–protein adducts can serve as a biomarker for the assessment of the severity of PA-ILI. However, the limitation of the study was that all PAs present in the PA-containing herbs tested in this study were retronecine-type PAs. Further investigations are certainly required into employing PAs of other types, such as otonecine-type PAs, in order to fully validate the use of hepatic pyrrole–protein adduct formation as a biomarker of PA-ILI.

More than 660 PAs have been found in up to 6000 plant species that may be used as herbal preparations and/or found as contaminants in various foodstuffs, such as wheat, tea, and honey [1,2,3]. Given the high prevalence of PA exposure through the consumption of these PA-containing products, several regulations to limit human PA intake have been established by food and/or drug administration authorities in different countries, such as the Australia New Zealand Food Authority, the British Committee on Toxicity of Chemicals in Food, the German Federal Institute for Risk Assessment and the European Food Safety Authority. However, these precautionary regulations usually assume that all PAs possess similar toxic potency as the most potent PAs, i.e., lasiocarpine or riddelliine, but it has been demonstrated that structurally different PAs might have significantly different toxicities [33,34]. Moreover, humans are generally exposed to multiple PAs via the consumption of PA-containing or PA-contaminated products. Therefore, an evaluation of the toxicities of structurally different PAs, individually or in a mixture, will give important information for setting up more appropriate regulations. In the present study, we demonstrated, for the first time, a significant and direct correlation between the level of hepatic pyrrole–protein adducts formed and the PA-induced elevation of serum ALT activity in rats, as evidenced by the good linearity in their correlation (Figure 5). Furthermore, unlike in vitro studies which compare the toxic potencies of different PAs in cultured cells, we used an animal model with the involvement of absorption, distribution, metabolism, and excretion processes of PAs, all of which have been proven to significantly affect PA intoxication [26,35]. Therefore, the hepatotoxic potencies of different PAs or their mixtures could be more accurately assessed in the animal model. Apart from the animal model, hepatotoxic potencies of different PAs or their mixtures can also be quickly assessed based on the formation of pyrrole–protein adducts in the in vitro hepatic microsomal incubation. Emerging evidence indicates that the formation rate of pyrrole–protein adducts, as an indicator of PA metabolic bioactivation, demonstrates a clear structure–toxicity relationship of structurally diverse PAs [6,7,36]. For instance, the diester retronecine-type PAs (such as lasiocarpine), which produce the highest amount of pyrrole–protein adducts, exert the highest hepatotoxic potency; these are followed by monoester retronecine-type PAs, which form relatively fewer amounts of pyrrole–protein adducts, and exert lower hepatoxic potency; meanwhile, platynecine-type PAs, which do not generate pyrrole–protein adducts, are generally non-toxic [6,7]. Considering that plants and foodstuffs normally contain more than one PA in a mixed form, the determination of the total pyrrole–protein adducts derived from all toxic PAs provides a new insight into the evaluation of the hepatotoxic potency of PA mixtures. With improved knowledge on the health hazard that is induced by hepatic pyrrole–protein adducts and exposure to PA, the hepatotoxic dose of PAs or PA-containing natural products can be rapidly determined.

Clinically, the diagnosis of drug-induced liver injury (DILI) and herb-induced liver injury (HILI) is always a major challenge, due to the lack of reliable biomarkers for use in general clinical practice. Recently, the Nanjing criteria, an expert consensus on the diagnostic criteria of PA-induced HSOS, was announced by the Hepatobiliary Diseases Committee of the Chinese Society of Gastroenterology [37]. The Nanjing criteria relies mainly on: (1) A history of ingesting a PA-containing plant and excluding other causes of liver injury; (2) Abdominal distention and/or pain in the hepatic region, hepatomegaly, and ascites; (3) Elevation of serum total bilirubin or abnormal liver function testing; 4) Typical features of enhanced Computed Tomography and Magnetic Resonance Imaging. However, in most cases, patients cannot provide a clear intake history of PA-containing herbs/health supplements, and imaging examinations are considered as complementary methods with limited specificity. The Roussel Uclaf Causality Assessment Method (RUCAM), especially the updated RUCAM [38], provides a robust quantitative approach to assess the causality of suspected DILI and HILI cases. A recent comprehensive review illustrated the wide use of the RUCAM for causality assessment in 81,856 idiosyncratic DILI and 14,029 HILI cases; among them, there were 28 PA-induced HSOS cases [39]. In China, the RUCAM is also recommended in the Chinese Society of Hepatology (CSH) guidelines for the diagnosis and treatment of DILI [40]. Increasing evidence suggests that PA exposure biomarkers, such as pyrrole–protein adducts, pyrrole–DNA adducts, and pyrrole–amino acid adducts, are specific and applicable diagnostic biomarkers. This evidence supports the causality assessment used in the updated RUCAM [27,41,42,43]. Therefore, for future clinical diagnosis of suspected PA-induced HSOS cases, the RUCAM, the Nanjing criteria, and specific PA exposure biomarkers should be applied together to confirmatively diagnose PA-induced HSOS cases.

## 4. Materials and Methods

### 4.1. Chemicals

Monocrotaline, trifluoroacetic acid, 4-dimethylaminobenzaldehyde, silver nitrate, and 60% perchloric acid were purchased from the Sigma Chemical Co. (St. Louis, MO, USA). Senecionine and seneciphylline were bought from ChromaDex Co. (Irvine, CA, USA). Ethanolic silver nitrate was prepared by dissolving 1 g silver nitrate in 50 mL absolute ethanol containing 5% trifluoroacetic acid. Ehrlich reagent was made by adding 1 g 4-dimethylaminobenzaldehyde and 0.7 mL 60% perchloric acid into 50 mL absolute ethanol.

### 4.2. Plant Materials

Roots of *C. sessiliflora* (monocrotaline-containing herb) and *G. japonica* (seneciphylline- and senecionine-containing herb) were collected in Hubei and Anhui Province in China, respectively, and authenticated according to conventional pharmacognosy procedures [44]. The herbal aqueous extracts were prepared according to our previously developed extraction method [45,46]. Briefly, individual herbs were dried, chopped into small pieces, and refluxed with distilled water (1:6, *w/w*) for 1 h. The same extraction procedure was repeated two more times. The aqueous extracts were combined and centrifuged at 3000× *g* for 10 min. The supernatant was dried under reduced pressure to give syrup, further lyophilized to yield dried powders, and then stored at −20 °C for further studies. The yields for *G. japonica* and *C. sessiliflora* extract were 30% and 8%, respectively. Monocrotaline (0.64 mmol/g extract) in *C. sessiliflora* extracts and senecionine (8.37 nmol/g extract) and seneciphylline (17.71 nmol/g extract) in *G. japonica* extracts were identified and quantified using our previously developed HPLC–UV–MS method [45]. 

### 4.3. Treatment of Rats with Herbal Extracts

Procedures involving the care and handling of the rats were reviewed and approved by the Animal Experimental Ethics Committee, The Chinese University of Hong Kong (CUHK), under the regulations of the Hong Kong SAR government. Male Sprague-Dawley (SD) rats (190–220 g) obtained from the Laboratory Animal Services Centre of CUHK were housed in a controlled environment (25 °C, 50% relative humidity and 12 h light–dark cycle) and allowed free access to standard chow and water. Rats were randomly divided into various dose groups (*n* = 5/group) and orally administered with a single dose of *C. sessiliflora* extract at doses ranging from 0.50 g/kg to 1.5 g/kg (equivalent to monocrotaline of 0.32 to 0.96 mmol/kg), or *G. japonica* extract at doses ranging from 3 g/kg to 18 g/kg (equivalent to total PA of 0.078 to 0.47 mmol/kg). Moreover, to confirm that hepatotoxicity was induced primarily by PA, a single dose of monocrotaline (0.80 mmol/kg) was orally given to rats for a direct comparison with the results from monocrotaline-containing *C. sessiliflora* extract. The vehicle control groups were conducted in parallel by dosing rats with distilled water. At 24 h after administration, all treated rats were sacrificed, and the livers and blood samples were collected for the examination of serum ALT, hepatic GSH, hepatic pyrrole–protein adducts, and histological changes. 

### 4.4. Biochemical Assays and Histological Determination

The activity of serum ALT and hepatic levels of GSH were determined with the standard spectrophotometric methods previously described [17,30]. Histological changes, including both hemorrhage and coagulative necrosis, in the livers of PA-treated rats were examined by standard hematoxylin–eosin (H&E) staining procedures [30]. 

### 4.5. Detection of Pyrrole–Protein Adducts in Liver Samples

To develop a more practical, simple, rapid, and cost-effective method for future application as a rapid screening method, a well-established spectrophotometric method [13,46] was adapted to measure hepatic pyrrole–protein adducts. Briefly, 1 g liver portion was homogenized, after dry blotting, in 10 mL acetone and centrifuged at 900× *g* for 5 min. The residue was washed with 10 mL absolute ethanol and centrifuged for 5 min, and the resulting residue was reconstituted in 5 mL ethanolic silver nitrate. The mixture was shaken for 30 min, followed by centrifugation at 900× *g* for 5 min. The supernatant was collected. The residue was washed with 5 mL ethanol and centrifuged, and supernatants were collected. Supernatants were combined, mixed with Ehrlich reagent (containing 4-dimethylaminobensaldrhyde) in the ratio of 4:1 (*v/v*), and heated at 55 °C for 10 min to generate a pyrrole-derived analyte (Figure 2). The absorbance of the analyte was measured at 562 nm and 625 nm on the Pharmacia-LKB 4060 UV-VIS spectrophotometer. An adjusted absorbance (A) was determined from the formula A = 1.1 × (A_562_ − A_625_) to correct for the endogenous absorption at 625 nm. The amount of pyrrole–protein adducts was calculated using a molar absorptivity of 60,000 [13,46]. 

### 4.6. Statistical Analysis

Data were expressed as mean ± SEM (*n* = 5). One-way ANOVA (analysis of variance) and Dunnett’s multiple comparison post-test were used for comparison among three or more groups. Student’s *t*-test was used for comparison between two groups. Statistical significance was set at *p* < 0.05.

## Figures and Tables

**Table 1:** Levels of serum ALT, hepatic GSH, and pyrrole–protein adducts in rats dosed with *Crotalaria sessiliflora* and *Gynura japonica* water extracts and monocrotaline at 24 h after administration.

Dose of Herbal Extract (g/kg)	Equivalent to PA Dose (mmol/kg)	Pyrrole–Protein Adducts (nmol/g Liver)	ALT (SF unit/mL)	GSH (µmol/g Liver)	Animal Group
Different batches of control groups					
0	0	0	50.19 ± 2.21	4.25 ± 0.23	A
0	0	0	43.00 ± 2.89	7.70 ± 0.52	B
0	0	0	40.87 ± 1.28	5.30 ± 0.31	C
0	0	0	47.32 ± 1.96	4.15 ± 0.23	D
*Crotalaria sessiliflora* water extract					
0.50	0.32	6.46 ± 1.90	56.04 ± 3.12	7.91 ± 0.40	B
0.60	0.38	13.48 ± 2.11	57.04 ± 4.51	10.37 ± 0.42 **	B
0.62	0.40	17.78 ± 2.87	57.75 ± 6.26	6.05 ± 0.46	C
0.78	0.50	14.71 ± 1.59	56.64 ± 7.55	7.36 ± 0.16 **	C
0.80	0.51	16.90 ± 1.71	58.64 ± 8.52	11.14 ± 0.19 **	B
0.94	0.60	25.57 ± 2.64	57.54 ± 6.95	7.91 ± 0.26 **	C
1.00	0.64	20.74 ± 1.66	54.57 ± 6.05	8.85 ± 0.89 **	A
1.09	0.70	28.59 ± 0.91	71.81 ± 5.35 **	7.73 ± 0.43 **	C
1.20	0.77	26.97 ± 0.71	75.63 ± 4.30 *	9.85 ± 0.34 **	A
1.50	0.96	32.96 ± 2.35	88.59 ± 13.69 **	8.66 ± 0.51 **	A
Monocrotaline					
	0.80	29.55 ± 1.38	81.73 ± 11.04 *	7.52 ± 0.59 *	A
*Gynura japonica* water extract					
3	0.08	7.44 ± 1.05	52.36 ± 1.70	3.99 ± 0.37	D
6	0.16	16.24 ± 2.09	55.67 ± 3.28	5.66 ± 0.30	D
12	0.31	28.10 ± 3.11	61.98 ± 5.36	6.75 ± 0.74 **	D
15	0.39	30.88 ± 0.89	68.29 ± 7.68	10.19 ± 0.36 **	D
18	0.47	39.23 ± 1.90	83.34 ± 18.34 *	6.24 ± 0.64 **	D

Data are expressed as mean ± SEM (*n* = 5). SF unit means Sigma-Frankel unit. The experiments were separately conducted in four individual batches (group A, B, C, and D); thus, statistical analysis was conducted in individual experimental groups, respectively. Equivalent to PA doses were calculated by the content of PAs in individual herbal extracts. * *p* < 0.05, ** *p* < 0.01: compared with the corresponding control.

**Table 2:** Morphological changes in the livers of rats treated with *Crotalaria sessiliflora* water extract and monocrotaline, 24 h after treatment.

Dose of *Crotalaria* *sessiliflora* Water Extract (g/kg)	Equivalent to Monocrotaline Dose (mmol/kg)	Hemorrhage	Coagulative Necrosis
0.00	0.00	-	-
0.50	0.32	-	+
0.60	0.38	-	+
0.62	0.40	-	+
0.78	0.50	+	++
0.80	0.51	+	++
0.94	0.60	++	+++
1.00	0.64	++	+++
1.09	0.70	+++	+++
1.20	0.77	+++	+++
MCT *	0.80	+++	+++
1.50	0.96	+++	+++

Both parameters were graded on a four-point system. Hemorrhage: -, absent; +, mild (only a small area around the central vein involved); ++, moderate (most of the area around the central vein involved with centrilobular extension); +++, severe (most of the area of the centrilobular region involved with extension into mid-lobular region). Coagulative necrosis: -, absent; +, mild (centrilobular region involved but without mid-lobular extension); ++, moderate (some lobules involved with mid-lobular extension); +++, severe (most of lobules involved with mid-lobular extension). *: monocrotaline was orally administered.

## Data Availability

Data is contained within the article.

## Abbreviations

Pyrrolizidine alkaloids (PAs); PA-induced liver injury (PA-ILI); alanine aminotransferase (ALT); glutathione (GSH); dehydropyrrolizidine alkaloids (dehydro-PAs); drug-induced liver injury (DILI); herb-induced liver injury (HILI); hepatic sinusoidal obstruction syndrome (HSOS).
